# Genetic characterization of a group of commercial African timber species: From genomics to barcoding

**DOI:** 10.1371/journal.pone.0284732

**Published:** 2023-04-20

**Authors:** Maurizio Mascarello, Olivier Lachenaud, Mario Amalfi, Erik Smets, Olivier J. Hardy, Hans Beeckman, Steven B. Janssens

**Affiliations:** 1 Meise Botanic Garden, Meise, Belgium; 2 Department of Biology, KU Leuven, Leuven, Belgium; 3 Unity of Plant Epigenetics, Research and Innovation Centre, Fondazione Edmund Mach, San Michele all’Adige, Italy; 4 Herbarium et Bibliothèque de Botanique Africaine, Université Libre de Bruxelles (ULB), Brussels, Belgium; 5 Fédération Wallonie–Bruxelles, Service Général de l’Enseignement Universitaire et de la Recherche Scientifique, Brussels, Belgium; 6 Naturalis Biodiversity Center, Leiden, The Netherlands; 7 Evolutionary Biology and Ecology, Université Libre de Bruxelles (ULB), Brussels, Belgium; 8 Wood Biology, Department of Biology, Royal Museum for Central Africa, Tervuren, Belgium; Institute for Biological Research, University of Belgrade, SERBIA

## Abstract

In the last decades, illegal logging has posed a serious threat for the integrity of forest ecosystems and for biodiversity conservation in tropical Africa. Although international treaties and regulatory plans have been implemented to reduce illegal logging, much of the total timber volume is harvested and traded illegally from tropical African forest regions. As a result, the development and the application of analytical tools to enhance the traceability and the identification of wood and related products is critical to enforce international regulations. Among available techniques, DNA barcoding is a promising approach for the molecular identification of plant species. However, although it has been used successfully for the discrimination of animal species, no set of genetic markers is available for the universal identification of plant species. In this work, we firstly characterized the genetic diversity of 17 highly-valuable African timber species from five genera (*Afzelia*, *Guibourtia*, *Leplea*, *Milicia*, *Tieghemella*) across their distribution ranges in West and Central Africa using the genome skimming approach in order to reconstruct their chloroplast genomes and nuclear ribosomal DNA. Next, we identified single-nucleotide polymorphisms (SNPs) for the discrimination of closely-related species. In this way, we successfully developed and tested novel species-specific genetic barcodes for species identification.

## Introduction

Several initiatives have been developed in order to promote the conservation of tropical tree species by prohibiting the illegal trade of valuable timber [1]. These include the Convention on International Trade of Endangered Species of Wild Fauna and Flora (CITES, www.cites.org) and the International Union for Conservation of Nature (IUCN, www.iucn.org), as well as regulations such as the European Union Forest Law Enforcement, Governance and Trade (EU FLEGT) Action Plan (http://www.euflegt.efi.int) and the EU Timber Regulation (EUTR) [2]. In tropical areas, however, a huge amount of timber is still illegally harvested. This amount has been estimated to reach up to 90% of the total timber trade in some tropical countries such as the Democratic Republic of Congo [3–5]. Criminal organizations and logging companies apply different strategies to overcome the control of timber trade and export by local and international authorities, such as false claims of geographic origin, logging beyond concessions, falsification of export permits, and the amalgamation of legal and illegal timber [6–9].

The most common forensic approach for the identification of timber species in the international market is the microscopic analysis of wood products. Although this is usually sufficient to identify wood specimens at the genus level, this method is often inadequate to discriminate closely-related species which are characterized by similar wood anatomical features [10, 11]. A recently developed approach for plant identification is DNA barcoding, which makes use of conventional plant DNA barcodes for species discrimination. The most commonly used DNA barcodes are plastid regions such as the protein-coding genes *matK* and *rbcL*, the *trnL* intron, and the intergenic spacers *psbA-trnH* and *trnL-trnF*, as well as the nuclear ribosomal spacers ITS1 and ITS2 [12–15]. The high number of copies per cell of both the plastid genome and nuclear ribosomal DNA repeats increases the chance of successful DNA amplification using samples with degraded DNA, as often the case in wood products [16]. Unfortunately, although DNA barcoding improved the identification of plant species, it cannot be applied as a universal method for species-level identification of tropical trees [16, 17]. For these reasons, high-throughput sequencing (HTS) technologies have been recently used to search for novel genetic markers to improve plant species identification. For example, Song et al. [18] and Hong et al. [19] found candidate chloroplast genes for the identification of commercial South East Asian timber species belonging to the genera *Dalbergia* and *Pterocarpus*, respectively. In addition, Mascarello et al. [9] created a genetic reference database that contains the chloroplast genomes of 62 commercial tropical African timber species. In addition, Mascarello et al. [9] identified possible genetic regions in the plastome that could help to improve species identification. However, data from a single individual per species is not sufficient to produce robust plant barcodes to distinguish closely-related species, due to intraspecific genetic variation, which is often eco-geographically structured, and the risk of shared variation between species under incomplete lineage sorting. Furthermore, cases of chloroplast capture among sympatric individuals of closely-related species may occur, as found in the genera *Afzelia* [20], *Milicia* [21] and *Brachystegia* [22], so that regions from the nuclear ribosomal DNA, including ITS1 and ITS2, need to be analyzed in addition to plastid markers.

This work aims to analyze inter- and intraspecific genetic variation in plastid genes and nuclear ribosomal transcribed spacers in a group of commercially important tropical African timber species in order to identify reliable genetic markers for species identification. Firstly, we used HTS technologies and bioinformatics tools to assemble *de-novo* the chloroplast genome, also called plastome (ptDNA), and the nuclear ribosomal DNA (nrDNA) of the targeted species. Secondly, we performed a comparative genomic analysis to search for genetic regions suitable for the discrimination of closely-related species. Finally, we developed and tested primers for the PCR amplification and sequencing of the selected barcodes.

## Materials and methods

### Plant sampling

Leaf samples were obtained from herbarium specimens and silica-dried material from 17 commercial timber species in tropical Africa, belonging to the genera *Afzelia* (Fabaceae), *Guibourtia* (Fabaceae), *Leplaea* (Meliaceae), *Milicia* (Moraceae) and *Tieghemella* (Sapotaceae) (Tables 1 and S1). These species are listed in international agreements for the global conservation of animal and plant biodiversity such as the CITES, and the IUCN Red List. All essential permits have been obtained for the collection, export and research on samples collected after the amendment of the Nagoya Protocol for Access and Benefit-Sharing (www.cbd.int).

**Table 1:** Commercial African timber species analyzed in this work.

Family	Genus	Species
Fabaceae	*Afzelia*	*A*. *africana*, *A*. *bella* (*var*. *bella* and *var*. *gracilior*), *A*. *bipindensis*, *A*. *pachyloba*, *A*. *quanzensis*
	*Guibourtia*	*G*. *arnoldiana*, *G*. *demeusei*, *G*. *ehie*, *G*. *pellegriniana*, *G*. *tessmannii*
Meliaceae	*Leplaea*	*L*. *cedrata*, *L*. *laurentii*, *L*. *thompsonii*
Moraceae	*Milicia*	*M*. *excelsa*, *M*. *regia*
Sapotaceae	*Tieghemella*	*T*. *africana*, *T*. *heckelii*

### DNA extraction and high-throughput sequencing

Genomic DNA from dried leaf samples was isolated using the CTAB-based extraction protocol [9, 23]. DNA concentration (ng/μl), average fragment size (bp) and quality (GQN = 300 bp) were measured using Fragment Analyzer (Agilent, US).

DNA library preparation was performed through an initial enzymatic fragmentation to 200–450 bp using NEBnext® Ultra™ II FS DNA Library Prep Kit for Illumina® (New England Biolabs, US) and NEBNext® Multiplex Oligos for Illumina® (New England Biolabs, US). Highly-degraded DNA samples were processed without starting fragmentation using NEBnext® Ultra™ II DNA Library Prep Kit for Illumina® (New England Biolabs, US). DNA library double-size selection (320–470 bp) was done using SPRIselect® (Beckman Coulter, US). The average fragment size (bp) and molarity (nM) of the final products were calculated using Fragment Analyzer (Agilent, US).

DNA libraries were sent to RAPiD Genomics (Florida, US) and Fasteris SA (Plan-les-Ouates, Switzerland) for paired-end sequencing at low genome coverage sequencing (0.1-10X, 2x150 bp), aiming at a minimum of 5 million reads per sample, using HiSeq® 3000, as well as HiSeq® 4000 and NovaSeq® 6000 (Illumina, US).

### Genome assembly

Illumina sequencing reads were checked for quality control using FastQC [24]. Plastomes and nuclear ribosomal DNA contigs were *de-novo* reconstructed using the automated pipeline GetOrganelle [25], which depends on the software Bowtie2 [26], SPAdes 3 [27] and NCBI BLAST+ [28]. The reconstructed chloroplast genomes were aligned with the respective reference plastomes from GenBank (http://www.ncbi.nlm.nih.gov/genbank/) using MAFFT v.7 [29] in order to rearrange the orientation of flip-flop sequences using Geneious Prime [30]. The assembly of plastid contigs obtained from incomplete genome reconstructions was completed using Geneious Prime, supported by the alignment with the respective GenBank reference genome. ITS1, ITS2 and the nuclear ribosomal external transcribed spacer (ETS) were retrieved using Geneious Prime by mapping sequences available in GenBank.

### Genomic analysis and DNA barcoding

Plastomes and nuclear ribosomal contigs were aligned at the genus level using MAFFT v.7. Reference plastid genomes available in GenBank, published in Mascarello et al. [9], were included in the alignments (S1 Table). The search for *single nucleotide polymorphisms* (SNPs) was performed in Geneious Prime. Short polymorphic regions within selected plastid genes and nrDNA regions useful for species discrimination were developed and primers for the amplification of these regions were designed (S2 Table). All selected target regions exhibit a maximum length of 250 bp. The melting temperature and the GC content, as well as the formation of hairpins and primer self-dimerization were checked using Oligo Calc [31].

PCR amplification of the targeted regions using genomic DNA extracts from selected herbarium specimens was performed using KAPA2G Robust HotStart Polymerase (Roche, Germany). Thermocycler reactions were the same for all markers and set-up as follows: 1) Initial denaturation (95°C, 3–5 min), 2) 35 cycles of denaturation (95°C, 15 s), annealing (57°C, 20 s) and extension (72°C, 15 s), 3) Final extension (72°C, 1 min). Next, the amplified products were purified using Wizard® SV Gel and PCR Clean-Up System (Promega, US) and eluted with nuclease-free water. Then, the purified products were sent with the respective primers (5 μM) for Sanger sequencing to Macrogen (South Korea).

## Results

Using the Illumina sequencing technology in a ‘genome skimming’ manner, we retrieved high-quality sequencing reads from the high-copy chloroplast genome (ptDNA) and nuclear ribosomal DNA (nrDNA) of our 17 targeted African tree species. Plastid contigs were successfully *de-novo* assembled and identified by comparison with reference plastomes available in GenBank (S1 Table and S1 File). Nuclear ribosomal DNAs were also successfully *de-novo* reconstructed from Illumina sequencing reads (S2 File) and identified by mapping to available sequences downloaded from GenBank. Plastome and nuclear ribosomal contigs were further aligned at the genus level to assess inter- and intraspecific variation, which is important to further select genetic markers for species identification. The genetic identification of each specimen in this work involved a taxonomic check of botanical characters. More importantly, each target region (mini-barcodes) was also successfully amplified and sequenced upon the development of genus- or species-specific primers (S2 Table and S3 File).

### Afzelia

We focused on the commercial timber species of the genus *Afzelia*, which includes the savannah species *A*. *africana* and *A*. *quanzensis*, as well as the rainforest species *A*. *bella*, *A*. *bipindensis* and *A*. *pachyloba*. Furthermore, individual accessions of *A*. *bella* from both Central and West Africa (*A*. *bella var*. *bella* and *A*. *bella var*. *gracilior*, respectively) were also studied. We performed a multiple alignment of all individuals of the genus to target species-specific SNPs in the plastid genic regions (CDSs and introns) and in the nuclear ribosomal spacers. We were not able to search for polymorphic sites in the plastid intergenic spacers presenting hypervariable AT-rich microsatellites regions, such as *atpH-atpI*, *psbZ-trnG*, *rps4-trnT-trnL*, *rbcL-accD*, *rps3-rps19*, etc.

The genetic analysis of the ptDNA (S4 File) allowed the discrimination of individuals of *A*. *africana* and *A*. *quanzensis*, which present species-specific SNPs to discriminate against the other *Afzelia* species in this study (Tables 2 and 3). On the contrary, we did not observe any species-specific SNPs or indels in both genic and non-genic (intergenic spacers) regions among the rainforest *Afzelia* species in Central Africa (*A*. *bella var*. *bella*, *A*. *bipindensis*, *A*. *pachyloba*). Interestingly, we detected 11 polymorphic sites in the chloroplast genes to discriminate the Western variety of *A*. *bella* from the Central African one, eight of them located in the CDS regions (Tables 2 and 3).

**Table 2:** SNP variation in plastid CDS regions among *Afzelia* species. “Central African rainforest species” indicates *A*. *bella var*. *bella*, *A*. *bipindensis* and *A*. *pachyloba*.

*A*. *africana*	/			
*A*. *quanzensis*	94	/		
*A*. *bella var*. *gracilior*	77	20	/	
Central African rainforest species	80	20	8	/
	*A*. *africana*	*A*. *quanzensis*	*A*. *bella var*. *gracilior*	Central African rainforest species

**Table 3:** SNP variation in plastid intron regions among *Afzelia* species. “Central African rainforest species” indicates *A*. *bella var*. *bella*, *A*. *bipindensis* and *A*. *pachyloba*.

*A*. *africana*	/			
*A*. *quanzensis*	25	/		
*A*. *bella var*. *gracilior*	20	5	/	
Central African rainforest species	22	8	3	/
	*A*. *africana*	*A*. *quanzensis*	*A*. *bella var*. *gracilior*	Central African rainforest species

The analysis of the nrDNA (S5 File) also shows several polymorphic sites to distinguish *A*. *africana* and *A*. *quanzensis* (Tables 4 and 5), as well as frequent double-nucleotide polymorphisms (DNPs), but no species-specific SNPs/DNPs or indels among the rainforest *Afzelia* species in Central Africa were found. Individuals of *A*. *bella* var. *gracilior* do not exhibit SNPs/DNPs in the ITS1 and ITS2 to discriminate against the Central African variety, while we only detected three putative polymorphic sites in the external transcribed spacer (ETS) to distinguish the two varieties.

**Table 4:** SNP variation in the ETS region among *Afzelia* species. “Central African rainforest species” indicates *A*. *bella var*. *bella*, *A*. *bipindensis* and *A*. *pachyloba*.

*A*. *africana*	/			
*A*. *quanzensis*	11	/		
*A*. *bella var*. *gracilior*	24	28	/	
Central African rainforest species	18	20	3	/
	*A*. *africana*	*A*. *quanzensis*	*A*. *bella var*. *gracilior*	Central African rainforest species

**Table 5:** SNP variation in the ITS (ITS1 & ITS2) region among *Afzelia* species. “Central African rainforest species” indicates *A*. *bella var*. *bella*, *A*. *bipindensis* and *A*. *pachyloba*.

*A*. *africana*	/			
*A*. *quanzensis*	14	/		
*A*. *bella var*. *gracilior*	34	34	/	
Central African rainforest species	24	26	/	/
	*A*. *africana*	*A*. *quanzensis*	*A*. *bella var*. *gracilior*	Central African rainforest species

We observed that the specimen *A*. *bella var*. *gracilior* “Jongkind 9400”, collected in Liberia, is characterized by 21 different polymorphic sites in the nuclear ribosomal spacers compared to the other specimens of *A*. *bella var*. *gracilior* processed in this study. Therefore, we sequenced the ptDNA and the nrDNA of a sample of *A*. *parviflora* (a related species not of commercial importance) from that region. Interestingly, we found that the nrDNA of *A*. *parviflora* is very close to that of the voucher “Jongkind 9400”. On the other hand, we did not find any SNP in the plastid genome for the discrimination between the two species. Because of the lack of sufficient botanical material, we could not judge if the sample “Jongkind 9400” was misidentified.

We successfully tested short DNA barcodes in the chloroplast CDSs to discriminate *Afzelia* species (S2 Table and S3 File). We targeted mini-barcodes in the coding regions of the *rpoC1* and *ndhF* genes exhibiting two and three SNPs, respectively, unique for *A*. *africana*. In addition, a barcode within the coding region of the *accD* gene included two SNPs that were unique for *A*. *quanzensis*. To discriminate the West African variety of *A*. *bella* from the Central African rainforest species, we amplified two short markers in the *rbcL* coding region, each one presenting one SNP variation. We also targeted a partial region of the ETS region to discriminate all species, except for those occurring in the Central African rainforest (S2 Table and S3 File).

### Guibourtia

We processed individuals belonging to the main commercial *Guibourtia* species in Central Africa (*G*. *arnoldiana*, *G*. *demeusei*, *G*. *ehie*, *G*. *pellegriniana*, *G*. *tessmannii*) to search for genetic variation useful for species identification. We also focused on the analysis of the intraspecific variation between individuals of *G*. *ehie* collected in the rainforests of Central and West Africa. As for the genus *Afzelia*, we did not manage to target polymorphic sites among the plastid intergenic spacers due to the occurrence of hypervariable AT-rich microsatellite regions.

The genetic comparison of the ptDNA (S6 File) shows a high number of SNPs in chloroplast genes (292 in the CDSs and 122 in the introns) between *G*. *demeusei* and *G*. *ehie* (Tables 6 and 7). These two species also exhibit a high number of polymorphic sites to discriminate against *G*. *pellegriniana* and *G*. *tessmannii* (Tables 6 and 7). On the contrary, the frequency of SNPs sites is much lower between *G*. *pellegriniana* and *G*. *tessmannii*, which present only 51 variations in the coding regions and 11 in the intron regions. Interestingly, we detected 84 SNPs in plastid genes (of which 54 located in the CDSs and 30 in introns) to discriminate between the Central and the West African individuals of *G*. *ehie*. We sequenced five individuals from Gabon, originally identified as *G*. *arnoldiana*, and did not observe either polymorphisms or indels to discriminate against Central African individuals of *G*. *ehie*. However, a re-examination of the herbarium material led to the conclusion that these five individuals belong to *G*. *ehie*, instead of *G*. *arnoldiana* (see Discussion).

**Table 6:** SNP variation in the plastid CDS regions among *Guibourtia* species.

*G*. *demeusei*	/			
*G*. *ehie*	292	/		
*G*. *pellegriniana*	305	346	/	
*G*. *tessmannii*	318	355	51	/
	*G*. *demeusei*	*G*. *ehie*	*G*. *pellegriniana*	*G*. *tessmannii*

**Table 7:** SNP variation in the plastid intron regions among *Guibourtia* species.

*G*. *demeusei*	/			
*G*. *ehie*	122	/		
*G*. *pellegriniana*	123	105	/	
*G*. *tessmannii*	124	104	11	/
	*G*. *demeusei*	*G*. *ehie*	*G*. *pellegriniana*	*G*. *tessmannii*

The genetic comparison of nuclear ribosomal spacers (S7 File) shows a high number of SNP variations among *Guibourtia* species (Tables 8 and 9), as well as several multi-nucleotide polymorphisms of different lengths. Interestingly, we also found 11 SNPs variations (of which six located in the ETS) between the individuals of *G*. *ehie* occurring in Central and West Africa. Furthermore, four SNPs variations in the ETS region occur between individuals of *G*. *pellegriniana* collected in Gabon and those from the Republic of the Congo. As expected, we did not observe any nucleotide variation between the samples misidentified as *G*. *arnoldiana* and individuals of *G*. *ehie* from Central Africa.

**Table 8:** SNP variation in the ETS region among *Guibourtia* species.

*G*. *demeusei*	/			
*G*. *ehie*	27	/		
*G*. *pellegriniana*	33	34	/	
*G*. *tessmannii*	33	32	16	/
	*G*. *demeusei*	*G*. *ehie*	*G*. *pellegriniana*	*G*. *tessmannii*

**Table 9:** SNP variation in the ITS (ITS1 & ITS2) region among *Guibourtia* species.

*G*. *demeusei*	/			
*G*. *ehie*	22	/		
*G*. *pellegriniana*	34	33	/	
*G*. *tessmannii*	31	28	17	/
	*G*. *demeusei*	*G*. *ehie*	*G*. *pellegriniana*	*G*. *tessmannii*

We tested mini-barcodes in the chloroplast coding regions to discriminate *Guibourtia* species (S2 Table and S3 File). We successfully targeted mini-barcodes in the coding regions of the *psbA* and *rpoC2*, to discriminate *G*. *pellegriniana* and *G*. *tessmannii* from *G*. *demeusei* and *G*. *ehie*. Among coding regions, the *rpoB* gene is the most SNP-rich to distinguish between *G*. *pellegriniana* and *G*. *tessmannii*, which exhibit six nucleotide variations. We were able to target a short barcode in the *rpoB* gene presenting two SNPs variations between *G*. *pellegriniana* and *G*. *tessmannii*. We also successfully amplified short regions in the CDS of *accD*, *psbE* and *ndhG* for the identification of *G*. *ehie* (S2 Table and S3 File). Furthermore, we retrieved a region in the *atpA* CDS exhibiting two SNPs to discriminate West African specimens of *G*. *ehie* against those of Central Africa. Finally, we amplified partial regions of ETS to discriminate all species (S2 Table and S1 File).

### Leplaea

The genus *Leplaea* (formerly included in *Guarea*) includes three important timber species in the tropical African rainforests (*L*. *cedrata*, *L*. *laurentii* and *L*. *thompsonii*). We processed samples of *L*. *cedrata* collected from both Central and West Africa. The analysis of the ptDNA (S8 File) and the nrDNA (S9 File) led to the identification of species-specific polymorphic sites to discriminate all *Leplaea* species (Tables 10–13). *Leplaea cedrata* exhibits a total of 223 and 190 SNPs in the plastome to discriminate against *L*. *laurentii* and *L*. *thompsonii*, respectively, while the two latter species only present 13 different polymorphic sites suitable for species identification in the chloroplast genome.

**Table 10:** SNP variation in plastid CDS regions among *Leplaea* species.

*L*. *cedrata*	/		
*L*. *laurentii*	92		
*L*. *thompsonii*	83	4	
	*L*. *cedrata*	*L*. *laurentii*	*L*. *thompsonii*

**Table 11:** SNP variation in plastid intron regions among *Leplaea* species.

*L*. *cedrata*	/		
*L*. *laurentii*	32		
*L*. *thompsonii*	25	4	
	*L*. *cedrata*	*L*. *laurentii*	*L*. *thompsonii*

**Table 12:** SNPs variations in the ETS region among *Leplaea* species.

*L*. *cedrata*	/		
*L*. *laurentii*	47		
*L*. *thompsonii*	43	8	
	*L*. *cedrata*	*L*. *laurentii*	*L*. *thompsonii*

**Table 13:** SNPs variations in the ITS (ITS1 & ITS2) region among *Leplaea* species.

*L*. *cedrata*	/		
*L*. *laurentii*	38		
*L*. *thompsonii*	32	7	
	*L*. *cedrata*	*L*. *laurentii*	*L*. *thompsonii*

In *L*. *cedrata*, we found that the individuals collected in the East DRC, Cameroon, Republic of Congo, and Guinea, exhibit 164 different polymorphic sites (of which 66 in the CDSs and 19 in the introns) from the individuals occurring near the coast in Mayombe (DRC), Gabon and Ghana. On the contrary, we did not observe a high number of intraspecific polymorphic variation among these individuals in the nuclear ribosomal spacers.

We found two individuals of *L*. *laurentii* (vouchers “Harris 10090” and “Senterre 3591”) which have the same genotype as *L*. *thompsonii* in both chloroplast genes and nuclear ribosomal spacers. These collections unfortunately lack flowers and fruits, although they match *L*. *laurentii* in vegetative characters (see Discussion).

We selected short plastid barcodes for the identification of *Leplaea* species (S2 Table and S3 File). We successfully amplified the intergenic spacer *rpoC2-rpoC1* and short sequences in the *rpoC1*, *rbcL* and *accD* coding regions, which exhibit SNPs for the identification of *L*. *cedrata*. The *rbcL* gene also presents three intraspecific polymorphic variations in *L*. cedrata. Furthermore, the mini-barcodes in the *rbcL* and the *accD* genes also exhibit one polymorphic site for the discrimination between *L*. *laurentii* and *L*. *thompsonii*. We also targeted a short region in ETS and ITS2 presenting various polymorphic sites for the distinction of all the three *Leplaea* species (S2 Table and S3 File).

### Milicia

We searched for genetic markers for the discrimination of the two *Milicia* species, *M*. *excelsa* and *M*. *regia*. We analyzed individuals collected in Western African regions in which the two species are either isolated or sympatric (Ghana, Ivory Coast). Furthermore, we also processed individuals of *M*. *excelsa* collected in Central Africa (S1 Table). The genetic comparison of the complete ptDNA (S10 File) shows that the two species differ only in 8 polymorphic sites, four of them located in coding regions and two occurring in intron regions. In the nrDNA (S11 File), ITS1 and ITS2 have a lower discriminative power (2 and 1 SNPs, respectively) while ETS presents a discrete number of polymorphisms (11 SNPs). The individuals of *M*. *excelsa* collected in Central Africa exhibit 36 different polymorphic sites (of which 13 in the CDSs and three in the introns) in the ptDNA from the West African individuals, while no variation in the nuclear ribosomal spacers was observed.

We successfully amplified four short barcodes in the chloroplast coding regions and two located in ETS (S2 Table and S3 File). We found that one individual of *M*. *regia* (voucher “Leeuwenberg 2471“) collected in Ivory Coast exhibits the polymorphisms of *M*. *excelsa* in the chloroplast genes. On the contrary, the ETS region presents the SNPs of *M*. *regia*. We confirmed after visual inspection of the reference material that the specimen was correctly identified as *M*. *regia*. As a result, only the nuclear ribosomal spacers can be used for the genetic identification of *Milicia* species (see Discussion).

### Tieghemella

We evaluated the genetic variability between *Tieghemella africana and T*. *heckelii*, species respectively occurring in Central Africa and West Africa. *Tieghemella* species exhibit a high pairwise similarity in the chloroplast genome (99.9%), and they are highly conserved at the intraspecific level, with values of pairwise similarity ranging from 99.97% and 100%. The two species exhibit 18 polymorphic sites in the chloroplast genome (S12 File), six of these in the coding regions, while none are located in introns. We also observed 13 SNPs (seven in the ETS region and six in the ITS region) and two double-nucleotide polymorphisms in nuclear ribosomal spacers (S13 File). We successfully tested four short barcodes in plastid coding regions (*psbA*, *rps2*, *rpoC1* and *rps15*), as well as a region in the ETS and ITS1 spacers (S2 Table and S3 File).

## Discussion

We sequenced and reconstructed the plastid genome (ptDNA) and the nuclear ribosomal DNA (nrDNA) of commercial timber species from five different angiosperm genera using a genome skimming approach. Then, we performed a comparative genomic analysis at the genus level, allowing us to successfully identify stable and reliable *single nucleotide polymorphisms* (SNPs) for species and subspecies identification at the level of plastid genes and intergenic spacers, as well as in nuclear ribosomal spacers (ITS1, ITS2 and ETS). Then, we successfully developed and sequenced short novel barcodes (mini-barcodes) for species identification from highly-degraded plant material. On the other hand, we also found that genome skimming and DNA barcoding have limitations in the search for genetic markers in some taxa which do not exhibit species-specific genetic variation among closely-related species.

### Afzelia

In the genus *Afzelia*, we observed that the conventional plastid markers cannot be used for species identification in most cases, as previously reported [9]. Furthermore, we found that hyper-variable AT-rich microsatellite regions in some intergenic spacers (described above) are located in the plastomes of Fabaceae species such as *Afzelia* and *Guibourtia*. We confirm what was reported in Mascarello et al. [9] about the unsuitability of these regions for the development of genetic markers due to excessive variation at both inter- and intraspecific levels.

We confirm that the savannah-dwelling species *A*. *africana* and *A*. *quanzensis* can be discriminated both between each other and against the other rainforest-dwelling *Afzelia* species using both chloroplast genes and nuclear ribosomal spacers, in accordance with Donkpegan et al. [20]. The genetic diversification of these species may be related to the geographical isolation and the occupation of habitats with different ecological conditions [20].

On the contrary, we confirm that the rainforest species occurring in Central Africa (*A*. *bella var*. *bella*, *A*. *bipindensis and A*. *pachyloba)* cannot be distinguished using the ptDNA and the ITS1/ITS2 regions, as previously reported [20] In this work, we searched for polymorphic variations in the ETS region as a novel marker for species identification, but this did not show sufficient resolution for the discrimination of these species. As a consequence, DNA barcoding has limitations in the discrimination of these species compared to chemical-based technologies such as DART-TOFMS, which allowed the successful discrimination between *A*. *bipindensis* and *A*. *pachyloba* [32].

Our results suggest a possible case of hybridization among Central African tropical *Afzelia* species, related to the fact that they are sympatric through their entire geographic distribution range [20]. However, we discarded the hypothesis of cross-hybridization since Donkpegan et al. [33] found that the *Afzelia* species in this study are monophyletic, based on SNPs variation detected using genotyping-by-sequencing. This suggests that the search for novel genetic markers in nuclear genes is crucial for the genetic identification of Central African *Afzelia* species, while the scarce resolution of plastid and nuclear ribosomal markers may be related to incomplete lineage sorting or to the fact that the speciation events occurring for these species may be rather recent. On the other hand, polymorphic variation in chloroplast genes may play a role in the identification of the provenance of Central African rainforest species, but the processing of a larger number of individuals is needed to confirm our hypothesis.

Surprisingly, we found that the West African variety of *A*. *bella* (var. *gracilior*) presents distinct polymorphic sites in the ptDNA compared to *A*. *bella* var. *bella* and the other closely-related Central African rainforest *Afzelia* species. Our results suggest that chloroplast genes may be representative of genetic variations at the intraspecific level as a result of geographic isolation or different ecological conditions. On the other hand, we found that nuclear ribosomal spacers are not suitable for the discrimination between the two varieties of *A*. *bella*. The comparative analysis shows that although these regions are rather conserved at the intraspecific level in *A*. *bella* var. *gracilior* (as well as in *A*. *africana* and *A*. *quanzensis*), they show a high number of indistinct nucleotide variations among the Central African rainforest species. As a consequence, this may represent a serious drawback for the development of genetic markers in these regions for the discrimination of the two *A*. *bella* varieties. The possibility that these varieties could actually represent separate species needs further investigation.

We sequenced the ptDNA and the nrDNA of *A*. *parviflora* because we found a possible misidentification of a specimen of *A*. *bella var*. *gracilior* (voucher “Jongkind 9400”). Although there is no evidence of the exploitation of *A*. *parviflora* in the international timber market, the species is sympatric with *A*. *bella var*. *gracilior* in some West African regions, and it presents similar wood properties to the latter species [34]. Therefore, the occasional mixing in trade of these two species cannot be excluded. As for the Central African wet forest species, we did not observe species-specific polymorphic variations in chloroplast genes between *A*. *bella var*. *gracilior* and *A*. *parviflora*, in accordance with Donkpegan et al., [20], suggesting a possible case of chloroplast capture or incomplete lineage sorting. On the other hand, we found distinct SNPs in the nuclear ribosomal spacers. However, because of the limited number of specimens tested in this study, as well as the lack of botanical characters for taxonomic identification in the absence of flowers, further investigation to confirm our findings is needed.

Interestingly, we observed that *A*. *africana* is characterized by a large number of polymorphic sites in chloroplast genes, being suitable for the development of mini-barcodes for the successful discrimination against the other species. On the contrary, *A*. *quanzensis* shares more polymorphic sites in chloroplast genes with the rainforest *Afzelia* species, resulting in a limited number of genetic markers in the plastome for the discrimination of this species. However, the nuclear ribosomal spacers show abundant SNPs variations in both savannah species, being the most suitable markers for the identification of this species. Our results are in accordance with those of Donkpegan et al. [20], hypothesizing that *A*. *quanzensis* and the rainforest *Afzelia* species may have received the chloroplast genome from a common ancestor and that they did not originate from *A*. *africana*.

### Guibourtia

We searched for novel polymorphic sites for the discrimination of the five main commercial *Guibourtia* timber species in Central Africa. We found that the three CITES-listed species (*G*. *demeusei*, *G*. *pellegriniana*, *G*. *tessmannii*), and the widely-distributed species *G*. *ehie*, can be easily distinguished using both plastid genes and nuclear ribosomal spacers. These findings are crucial for the enforcement of the CITES regulation for these species, which can hardly be distinguished using traditional wood anatomical characters. These data support the findings of Tosso et al. [35], who found that *Guibourtia* species are mostly monophyletic, based on the sequencing of the entire ptDNA. The large number of polymorphic variations found between *G*. *demeusei* and *G*. *ehie*, and those found among these two species and *G*. *pellegriniana/G*. *tessmannii*, also support the morphological classification, based on leaf anatomical characters, established by Tosso et al. [35]. The latter divided *G*. *demeusei*, *G*. *ehie* and *G*. *pellegriniana/G*. *tessmannii* into three distinct morphological groups. Furthermore, the genetic variation among these species also reflects the taxonomic classification, in which these three groups of species are classified in three distinct sub-genera, respectively *Guibourtia*, *Gorskia* and *Pseudocopaiva* [35].

The genetic variation between *G*. *pellegriniana* and *G*. *tessmannii* is lower at the plastid gene level, but sufficient for the development of mini-barcodes containing multiple polymorphic sites for the discrimination of the two CITES-listed species. Despite the limited genetic variability, it is unlikely to observe cases of hybridization/introgression or chloroplast capture between the two species, as they seem to have separate geographical ranges. *Guibourtia pellegriniana* is only known from the coastal areas from Northern Gabon (Estuaire region) to the Mayombe forest area, while *G*. *tessmannii* is only found further inland in Cameroon and Gabon [35].

We did not manage to find novel genetic markers to identify *G*. *arnoldiana*, because all samples of this species included in the analysis proved to be misidentified as *G*. *ehie*. Indeed, the two species have been frequently confused in Gabon because the main character used to separate them in the vegetative state, i.e. the presence or absence of translucent dots in the leaves, is unreliable. These dots, assumed to be a distinctive feature of *G*. *arnoldiana* [36], are in fact sometimes present in *G*. *ehie* as well. A more reliable character is the presence of foliaceous stipules (usually caducous) in *G*. *ehie*, which are always absent in *G*. *arnoldiana*, even on seedlings or young shoots. In the field, another useful character to discriminate both species is the colour of the bark, which is bright orange-red in *G*. *arnoldiana* and grey-brown in *G*. *ehie*. Furthermore, the two species also seem to have different ranges, with *G*. *ehie* occurring north of the Nyanga river and *G*. *arnoldiana* restricted to the Mayombe range south of this river. However, since the Nyanga region is little-explored botanically, it is not impossible that they may locally share the same area. Further investigations should clarify this and provide suitable material for genomic analysis of *G*. *arnoldiana*.

While we found low genetic variation between the two varieties of *A*. *bella* at the intraspecific level, we observed a high number of polymorphic variations in the chloroplast gene markers in *G*. *ehie* between individuals distributed in Central Africa and those occurring in West Africa, as well as some genetic variation in nuclear ribosomal spacers. These findings provide further evidence for the usefulness of chloroplast regions to discriminate conspecific individuals from different geographic locations. Furthermore, the high level of polymorphic variation could also correspond to the occurrence of the taxa in different habitats. In Central Africa, *G*. *ehie* mainly occurs in evergreen forests, while in West Africa the species is mainly distributed in semi-deciduous forests [37]. Strong differentiation between the Central and West African populations of *G*. *ehie* was also found in SSR markers [38]. Therefore, further research should be done to evaluate whether selective processes between the two geographic groups of *G*. *ehie* led to the separation into two different species.

We observed large genetic variation at the intraspecific level in the chloroplast genes in *Guibourtia demeusei*, which is related to different geographic locations rather than differences in morphological characters. In addition, we tested a large number of individuals of *Guibourtia ehie* in Central Africa, of which one (voucher “Breteler 10505”) exhibits large polymorphic variation in the plastome compared to the other samples. However, contrary to what was observed between the Central and the West African individuals of *G*. *ehie*, we observed scarce intraspecific variation in the nuclear ribosomal spacers among individuals of *G*. *demeusei* and *G*. *ehie* in Central Africa. This supports the idea that the large polymorphic variation in the chloroplast genome corresponds to different eco-geographical conditions, rather than to a clear species delimitation.

### Leplaea

This is the first study that focuses on the genetic characterization of commercial timber species of the genus *Leplaea* using a genome-scale approach. Their relevance in the international timber market should not be underestimated, since they produce valuable timber, known as “bossé”, which is considered an excellent substitute for *Khaya* and *Entandrophragma* species [39]. We tested individuals of the species *L*. *cedrata*, *L*. *laurentii* and *L*. *thompsonii*, occurring both in regions where they are isolated and sympatric. Because of its wider geographic distribution, we also selected specimens of *L*. *cedrata* collected from both Central and West Africa.

The genomic analysis of the ptDNA and the nrDNA revealed distinct polymorphic sites for the discrimination among the three species. *L*. *cedrata* exhibits several polymorphic sites in both chloroplast genes and ETS/ITSs useful for the development of highly-variable mini-barcodes to discriminate against the other two species. On the contrary, the interspecific variation between *L*. *laurentii* and *L*. *thompsonii* in plastid genes is much lower, so that the simultaneous use of multiple genetic markers is essential for a reliable distinction between these two species. Although we did not observe cases of chloroplast capture between *L*. *laurentii and L*. *thompsonii*, we cannot exclude this process because of both similar morphological features and the sympatric occurrence in some geographic areas. As a consequence, we always recommend the use of plastid markers in association with nuclear ribosomal markers for these species in timber forensic identification.

Our analysis shows that two individuals identified as *L*. *laurentii* presented the genotype of *L*. *thompsonii*. Morphologically, both samples match *L*. *laurentii* in vegetative characters, having dark glandular dots on the lower leaf surface, which are considered typical of this species [40] but they, unfortunately, lack reproductive material, which would allow a more secure identification. However, their ptDNA and the nrDNA are very similar to those of *L*. *thompsonii* collected in those regions. A possibility could be that, as in *Guibourtia*, the presence or absence of glands is actually not a fully reliable character, but this needs further investigation.

Based on the results obtained from the chloroplast genome, we could divide *L*. *cedrata* into two main groups: the first includes the individuals collected in the regions of Eastern DRC, Cameroon, Republic of Congo, and Guinea; the second comprises those collected in Southwestern DRC (Mayombe range), Gabon and Ghana. Contrary to what is observed in *Afzelia bella*, *Guibourtia ehie* or *Milicia excelsa*, these intraspecific polymorphisms do not separate Central African from West African populations, but appear to be related to distance from the sea: the first group includes individuals from inland areas and the second from coastal areas.

In contrast to most of the species analyzed in this paper, we observed that the ptDNA of *L*. *laurentii* is highly conserved, as we found a low number of intraspecific polymorphic variations throughout its entire geographic distribution. As a consequence, the application of plastid markers to identify the provenance of *L*. *laurentii* samples may be possible, but with scarce resolution.

### Milicia

Because of the unsustainable exploitation level of “iroko” in the international timber market, the genus *Milicia* has been frequently studied in order to evaluate the interspecific variability between *M*. *excelsa* and *M*. *regia*, as well as intraspecific variation among individuals from different geographic locations [21, 41, 42]. However, none of the previous articles evaluated the genetic variation between the two species through the sequencing of the entire ptDNA and nrDNA. In this study, we selected individuals of *M*. *excelsa* and *M*. *regia* from several locations in Central and West Africa.

We found that the two species present a low number of SNP variations for species discrimination through the whole ptDNA. On the other hand, the individuals of *M*. *excelsa* occurring in West Africa exhibit a major number of polymorphic variations from the Central African individuals, being genetically closer to *M*. *regia*. This is in accordance to what was found by Daïnou et al. [41]. The intraspecific variation in *M*. *excelsa* is additional evidence of the genetic variation of the chloroplast genes depending on geographic location.

Although we found few species-specific polymorphic sites in the plastid genome to distinguish the two species, we discovered one individual of *M*. *regia* collected in Ivory Coast (voucher “Leeuwenberg 2471“) exhibiting the genotype of *M*. *excelsa* in the chloroplast genes, but presenting the genotype of *M*. *regia* in the nuclear ribosomal spacers. Therefore, we suspect that there may be cases of chloroplast capture between *Milicia* species in their areas of sympatry, as hypothesized in Daïnou et al. [21]. As a result, we recommend using nuclear ribosomal markers for species discrimination in the genus *Milicia*, particularly when the geographic origin is unknown. On the other hand, since ETS/ITSs show very little intraspecific variation in both species, our results support the idea that chloroplast genes represent the best markers for the identification of the provenance in closely related species such as *M*. *excelsa* and *M*. *regia*, independent of the occurrence of case of chloroplast capture between species.

### Tieghemella

As for the genus *Leplaea*, no genomic studies on the genus *Tieghemella* were carried out so far. We selected this genus for our study since both *T*. *africana* and *T*. *heckelii* are endangered due to over-exploitation for their attractive and highly durable timber. The two species also have identical wood morphological features, as well as very similar chemical profiles so that they cannot be distinguished using chemical technologies for timber identification such as DART-TOFMS [43]. Moreover, none of the conventional plastid markers exhibit polymorphic variation to discriminate these species, as previously reported [9].

Our analysis shows that, although the two *Tieghemella* species exhibit a low number of SNPs in both the ptDNA and nuclear ribosomal spacers, they show sufficient genetic variation for species discrimination. The two species have mostly separate ranges, with *T*. *heckelii* occurring exclusively in West Africa [44] and *T*. *africana* mostly in Central Africa [45], but there are reports of *T*. *africana* from Ivory Coast [46] suggesting possible sympatry between the two species; further investigation is needed to clarify this point. As for *Leplaea laurentii*, both *Tieghemella* species exhibit very low intraspecific variation in the plastid genome, which may be attributed to their slow generation rate.

## Conclusion

This is one of the first studies aimed at the selection of novel genetic markers for the identification of tropical African timber species of high relevance in the international timber market. We provided further evidence about the efficiency of the genome skimming method to unravel little-known genetic regions of organelle genomes. This led to the detection of novel species-specific polymorphic variations and to the development of mini-barcodes for the amplification and sequencing of highly-degraded DNA. Mini-barcodes were successfully used for the identification of natural herbal products [47], and their use should be extended to the forensic identification of commercial wood samples to trace illegal logging activities in tropical Africa. Besides the use of plastid markers and ITSs, we also evaluated the resolution power of the ETS, which resulted in an excellent marker for the molecular identification of the majority of the species in this study. The nuclear ribosomal spacers presented a higher discrimination rate than plastid markers, being sometimes suitable for the identification of the geographic origin of a sample. However, we noticed that the ETS/ITSs were not always suitable for the target of intraspecific variations because of either the occurrence of putative non-significant mutations or the absence of polymorphic sites (e.g., in *Milicia excelsa*). On the other hand, the chloroplast genome was revealed to be an excellent marker for intraspecific variation determined by different geographic locations and/or ecological conditions, particularly for species such as *Afzelia bella*, *Guibourtia ehie*, *Leplaea cedrata* and *Milicia excelsa*. A deeper investigation is needed to evaluate the resolution level of the chloroplast genome for the identification of individual provenance in the case of tropical African tree species. In conclusion, we strongly promote the use of genome skimming to expand the global plant barcode for timber forensics and other DNA barcoding applications.

## Supporting information

S1 Table Species list, vouchers and citations. (XLSX)

S2 Table Primers sequences and respective target regions. (XLSX)

S1 File List of reconstructed plastome sequences from Illumina sequencing. (FASTA)

S2 File List of reconstructed nuclear ribosomal DNA sequences from Illumina sequencing. (FASTA)

S3 File List of reconstructed sequences from Sanger sequencing. (FASTA)

S4 File Plastomes alignment of *Afzelia* species. (FASTA)

S5 File Nuclear ribosomal DNA alignment of *Afzelia* species. (FASTA)

S6 File Plastomes alignment of *Guibourtia* species. (FASTA)

S7 File Nuclear ribosomal DNA alignment of *Guibourtia* species. (FASTA)

S8 File Plastomes alignment of *Leplaea* species. (FASTA)

S9 File Nuclear ribosomal DNA alignment of *Leplaea* species. (FASTA)

S10 File Plastomes alignment of *Milicia* species. (FASTA)

S11 File Nuclear ribosomal DNA alignment of *Milicia* species. (FASTA)

S12 File Plastomes alignment of *Tieghemella* species. (FASTA)

S13 File Nuclear ribosomal DNA alignment of *Tieghemella* species. (FASTA)
